# Methotrexate an Old Drug with New Tricks

**DOI:** 10.3390/ijms20205023

**Published:** 2019-10-10

**Authors:** Yosra Bedoui, Xavier Guillot, Jimmy Sélambarom, Pascale Guiraud, Claude Giry, Marie Christine Jaffar-Bandjee, Stéphane Ralandison, Philippe Gasque

**Affiliations:** 1 Unité Mixte de Recherche Processus Infectieux en Milieu Insulaire Tropical (PIMIT), INSERM U1187, CNRS 9192, IRD 249, Université de La Réunion—Plateforme Technologique CYROI—2, rue Maxime Rivière, 97490 Sainte-Clotilde, France; yosra.bedoui.bouhouch@gmail.com (Y.B.); jimmy.selambarom@univ-reunion.fr (J.S.); pascale.guiraud@univ-reunion.fr (P.G.); 2 Service de Rhumatologie, CHU La Réunion site Félix Guyon, Allée des Topazes, CS11021, 97400 Saint Denis de La Réunion, France; xavier.guillot@chu-reunion.fr; 3 Laboratoire de biologie, CNR associé des arbovirus, CHU La Réunion site Félix Guyon, Allée des Topazes, CS11021, 97400 Saint Denis de La Réunion, France; claude.giry@chu-reunion.fr (C.G.); marie-christine.jaffarbandjee@chu-reunion.fr (M.C.J.-B.); 4 Service de Rhumatologie—Médecine Interne, CHU Morafeno, Route d’Ivoloina 501, Toamasina, Madagascar; stephane_ralandison@yahoo.fr; 5 Pôle de Biologie, secteur Laboratoire d’Immunologie Clinique et Expérimentale de la zone de l’Océan Indien (LICE-OI), CHU La Réunion site Félix Guyon, Allée des Topazes, CS11021, 97400 Saint Denis de La Réunion, France

**Keywords:** methotrexate, arthritis, inflammation, alarmin, virus, chikungunya, rheumatoid arthritis, innate immunity, pharmacology

## Abstract

Methotrexate (MTX) is the first line drug for the treatment of a number of rheumatic and non-rheumatic disorders. It is currently used as an anchor disease, modifying anti-rheumatic drug in the treatment of rheumatoid arthritis (RA). Despite the development of numerous new targeted therapies, MTX remains the backbone of RA therapy due to its potent efficacy and tolerability. There has been also a growing interest in the use of MTX in the treatment of chronic viral mediated arthritis. Many viruses—including old world alphaviruses, Parvovirus B19, hepatitis B/C virus, and human immunodeficiency virus—have been associated with arthritogenic diseases and reminiscent of RA. MTX may provide benefits although with the potential risk of attenuating patients’ immune surveillance capacities. In this review, we describe the emerging mechanisms of action of MTX as an anti-inflammatory drug and complementing its well-established immunomodulatory activity. The mechanisms involve adenosine signaling modulation, alteration of cytokine networks, generation of reactive oxygen species and HMGB1 alarmin suppression. We also provide a comprehensive understanding of the mechanisms of MTX toxic effects. Lastly, we discussed the efficacy, as well as the safety, of MTX used in the management of viral-related rheumatic syndromes.

## 1. Introduction

Methotrexate (4-amino-10-methylfolic acid, MTX), an analog and antagonist of folic acid, is commonly used in the treatment of a wide range of malignant and non-malignant diseases [1].

Originally developed as an anticancer medication, MTX is currently the first-line disease-modifying anti-rheumatic drugs (DMARDs) in the treatment of rheumatoid arthritis (RA), juvenile idiopathic arthritis, and psoriasis, and is useful in inflammatory bowel diseases, multiple sclerosis, vasculitis, systemic lupus erythematosus and other connective tissue diseases, and transplantation due to its beneficial anti-inflammatory and immunomodulatory activities [1,2,3]. 

MTX has also prompted a growing interest in the treatment of viral mediated arthritis [4]. Many viruses—including old world alphaviruses, Parvovirus B19, hepatitis B/C virus (HBV/HBC), and human immunodeficiency virus (HIV)—are associated with arthritogenic diseases [5]. Chronic viral arthritis can clinically mimic RA and last for months to years [6]. Given pathogenic similarities with RA, MTX may provide benefits in the treatment of chronic viral associated rheumatic disorders, although the potential risk to compromise patients’ immune surveillance to prevent viral reactivation may need to be considered [7].

MTX is considered an essential medication by the World Health Organization (WHO) and is incontestably one of the pharmaceuticals greatest success stories as it found indications widely distinct from its original intention [8,9]. Despite the introduction of a number of effective biological agents for the treatment of autoimmune inflammatory diseases and mainly RA, MTX remains one of the most efficient and most commonly used therapies against which the efficacy of new DMARDs is judged [1]. MTX can be used both as first-line monotherapy in DMARD-naive patients [10], and as an anchor drug, in MTX-insufficient responders, in combination with other conventional or biological DMARDs to maximize therapeutic effects [11,12]. Low and more infrequent doses of MTX are used to treat inflammatory diseases and compared with the 5 g/week doses used in the treatment of malignancy, once-weekly administration of MTX at 10 to 25 mg provides optimal clinical outcomes in RA, the commonest prototype of low-dose MTX indications [13]. 

Fundamental mechanisms underlying the therapeutic effect of high doses MTX on malignant diseases are currently well established; MTX as a folate antagonist, competitively inhibits the activity of folate-dependent enzymes and synthesis of purine and pyrimidine required for DNA and RNA production in rapidly dividing malignant cells [14]. However, mechanisms by which low-dose MTX exerts its therapeutic effect in inflammatory disorders are not completely elucidated.

MTX is known to have highly favorable cost-effectiveness and efficacy/toxicity ratios but toxicity is still a concern. The potential adverse events associated with MTX attract considerable attention as they represent the main cause of drug withdrawal [15,16,17]. There are two broad subsets of MTX associated adverse events; Symptomatic but rarely life-threatening adverse events such as nausea, headaches, fatigue, mucositis and hair loss, and potentially life-threatening adverse events including cytopenias, interstitial lung disease (or MTX pneumonitis), and MTX related liver disease (fibrosis and cirrhosis) [8]. The precise mechanisms of MTX toxicity are still not clear. MTX mechanisms of action regarding efficacy and toxicity may be determined by either the same or different metabolic pathways.

This review will focus on the molecular mechanisms of action of MTX as an anti-inflammatory/immunomodulatory drug in order to further understand MTX therapeutic and toxic effects in inflammatory autoimmune disorders. We also review the efficacy and safety of MTX use in viral induced arthritis.

## 2. History

MTX, formerly known as amethopterin, is one of several folic acid antagonists originally synthesized in the 1940s for the treatment of malignancies and structurally designed to inhibit dihydrofolate reductase (DHFR), an essential enzyme for purine and pyrimidine synthesis in cell proliferation [18,19,20].

The rationale for the introduction of MTX for the treatment of RA was assumed on its capacity to inhibit inflammatory and proliferative response of connective tissue. The closely related antifolate aminopterin, a synthetic derivative of pterin, was shown to suppress exudative and proliferative changes in experimental formaldehyde arthritis [21]. Aminopterin was gradually replaced by MTX due to its less toxic nature and the first documented clinical use of MTX for the treatment of RA was in 1951 [22]. MTX clinical potential as a RA treatment was suggested by Gubner, after studying the effects of MTX in RA patients, and was confirmed by well designed, blinded, placebo controlled studies conducted during the 1980s [23]. RA patients treated with MTX demonstrated improved global assessments of disease activity, joint scores, and marked decreases in pain. Since then, the use of weekly low doses of MTX has expanded to involve additional inflammatory and autoimmune diseases. 

In 1986, MTX was licensed by the US Food and Drug Administration (FDA) for the treatment of RA [24].The US FDA first approved the use of MTX only in life-threatening neoplastic diseases, or in patients with psoriasis or RA with severe, recalcitrant, disabling disease which is not adequately responsive to other forms of therapy [25]. Based on the American College of Rheumatology and the European League Against Rheumatism (ACR/EULAR) recommendations, MTX should be started early in recent and/or undifferentiated arthritis evocative of RA [26], in order to prevent joint destruction and disability.

Improved understanding of the pathogenesis of RA led to the introduction of biologic treatment in 1998 [27] and despite the development of several targeted biologic treatments such as TNF blockers, MTX remains the cornerstone of RA treatment, alone or in combination [28].

Continuous efforts are devoted to derivatives of MTX in order to improve the pharmacological parameters of the parent MTX. MTX derivatives bearing dihydro-2H-1,4-benzothiazine or dihydro-2H-1,4-benzoxazine applied on human synovial cells and human peripheral blood mononuclear cells (hPBMC) have been reported to have enhanced antiproliferative activity and increased DHFR binding affinity compared with MTX. In vivo, benzothiazine and benzoxazine derivatives exhibited antirheumatic activity in a rat adjuvant arthritis model [29]. Similar activities were observed with MTX derivatives containing enantiomerically pure l-*erythro*- or l-*threo*-γ-fluoroglutamic acid [30]. Didodecyl-MTX in lipid nanocarrier was found to reduce inflammation when administered via the intra-articular route into rabbits [31]. An optimized conjugate of MTX and hyaluronic acid (HA) was assessed on human fibroblast-like synoviocytes (FLS) and a synovial sarcoma cell line (SW982) and proved to be more efficient than the parent compounds to retrieve synovial inflammation in rat models [32].

## 3. Pharmacokinetics of MTX

In the treatment of inflammatory autoimmune diseases, MTX is commonly administered orally as a single weekly dose. In clinical practice, treatment is initiated at the dose of 10 mg/week, with an increase of 5 mg every 2–4 weeks up to a maximum dose of 20–30 mg/week, depending on clinical response or intolerance [33,34]. The use of parenteral MTX, particularly in the form of subcutaneous (SC) injection, has recently gained great interest and is of greater benefit over the oral route. SC MTX, was shown to have greater clinical efficacy and improved tolerability compared to the oral form. SC MTX administration is currently recommended in cases of insufficient clinical response or intolerance with oral MTX [34,35]. In Juvenile Idiopathic Arthritis (JIA), MTX is proved to be efficient at the dose of 10 to 20 mg/m^2^.

**Figure 1:**
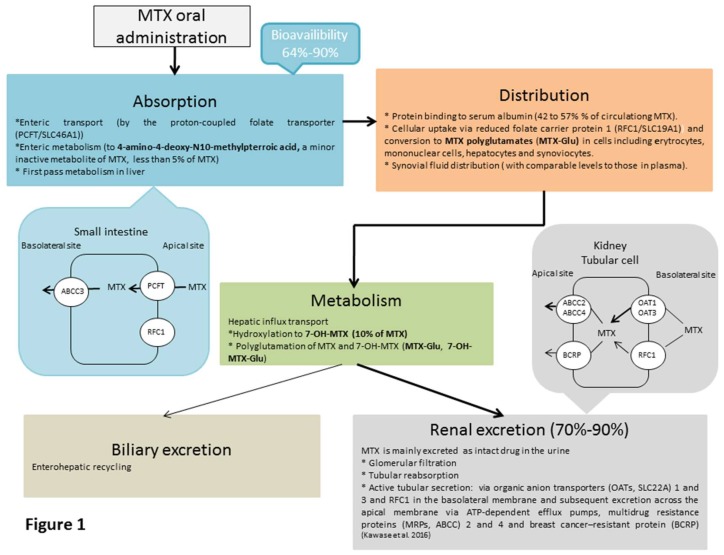
Pharmacokinetic process of MTX after oral administration. MTX absorption, distribution, metabolism, and excretion after oral administration.

After oral administration, MTX is absorbed in the proximal jejunum by the proton-coupled folate transporter (PCFT/SLC46A1), which transports reduced folates and MTX [36] (Figure 1). 

A modest fraction of MTX may be metabolized to 4-amino-4-deoxy-N10-methylpterroic acid through intestinal bacteria [37]. MTX bioavailability is relatively high, in the range of 64–90%. However, bioavailability varies widely among patients and decreases with increasing dose with a plateau effect at doses above 15 mg/week, suggesting saturation of the intestinal transporters [38,39,40]. MTX maximum plasma concentrations (Cmax) range between 0.3 and 1.6 µmol/L, and occur at a T max of 0.75 to 2 h after administration [37]. Several studies have demonstrated higher bioavailability with SC MTX than with oral MTX [38,39,41]. SC MTX injection will lead to a linear, dose-proportional increase in the blood circulation and no plateau effect [39]. Around 50% of circulating MTX is bound to plasma proteins [42,43,44,45]. MTX can distribute to the synovial fluid with comparable levels to those found in plasma [43]. MTX is subject to first-pass metabolism in the liver and is converted to 7-hydroxymethotrexate (7-OH-MTX), which is a major metabolite of MTX [46]. Renal excretion constitutes the major elimination route of MTX. The drug is filtered by the glomeruli, and additionally, undergoes active tubular secretion and reabsorption. A small proportion of MTX is excreted in the bile and some enterohepatic recycling also occurs [33,37,46,47]. The plasma half-life of low dose MTX varies from 4.5 h to 10 h [43,48]. MTX elimination is reduced in patients with impaired renal function, ascites, or pleural effusions. Such patients require especially careful monitoring for toxicity and require dose reduction or, in some cases, discontinuation of MTX treatment [45,48,49].

**Figure 2:**
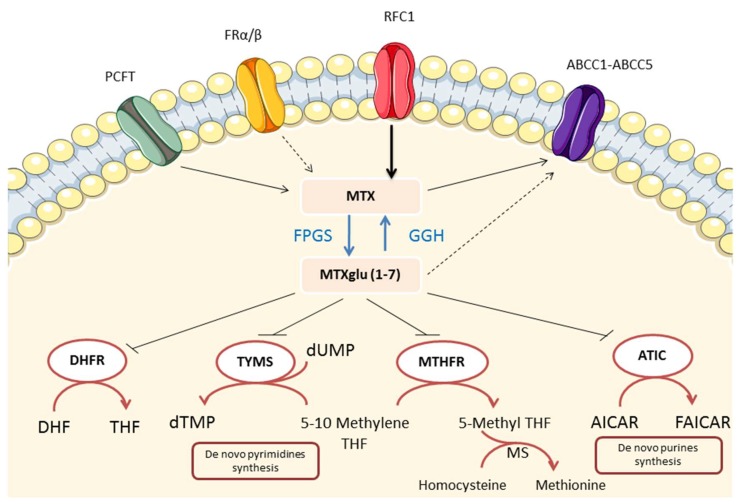
Methotrexate (MTX) transporters, metabolic pathways and intracellular enzyme targets. MTX transport across biological membranes is mediated by the reduced folate carrier (RFC1), the proton-coupled folate transporter (PCFT) (mainly expressed in the proximal part of the small intestine at the apical membrane of enterocytes) with limited contribution of folate receptors (FR). MTX is exported from the cell by the ATP-binding cassette (ABCC)-family transporters. Within the cell, MTX is converted to polyglutamate forms in a reversible reaction mediated by folylpolyglutamate synthetase (FPGS) and γ-glutamyl hydrolase (GGH). Polyglutamation reduces MTX efflux from the cell via ABCC transporters and therefore increases intracellular half-life of MTX. Intracellular formation of MTX polyglutamate also plays a critical role in MTX activity, increasing inhibition of dihydrofolate reductase (DHFR) and several folate dependent enzymes such as thymidylate synthase (TYMS), 5-amino-imidazole-4-carboxide ribonucleotide (AICAR) transformylase (ATIC), and methylene tetrahydrofolate reductase (MTHFR) decreasing downstream folate pathway intermediates required for nucleotide synthesis. DHF, dihydrofolate; THF, tetrahydrofolate; FAICAR, formyl AICAR; MS, methionine synthetase; dTMP, deoxythymidine monophosphate.

Cellular uptake of MTX is mediated by reduced folate carrier protein 1 (RFC1/SLC19A1), with limited contribution from the α and β folate receptors [33,36,50] (Figure 2). 

Cellular efflux of MTX is governed by the ATP-binding cassette proteins (ABCC), which play an important role as transporters for a large number of drugs and chemotherapeutic agents [36]. Within the cell, a proportion of intracellular MTX is converted to MTX polyglutamates by the catalytic action of folylpolyglutamate synthetase (FPGS), which adds up to seven glutamate residues to MTX [51,52]. MTX derivatives with chains longer than three glutamate residues are not substrates for the ABCC exporter proteins and have therefore enhanced cellular retention [51,53]. Polyglutamation is reversed by a deglutamation process, which is catalyzed by the γ-glutamyl hydrolase (GGH), producing a steady-state of intracellular MTX level [53,54]. MTX-polyglutamates are found in red blood cells, neutrophils, mononuclear cells, hepatocytes, and synoviocytes after oral administration [55]. Intracellular accumulation of MTX polyglutamates results in sustained efficacy of the drug and allows once weekly MTX administration in spite of its relatively short plasma elimination half-life [37,51]. MTX polyglutamates are believed to be the active form of the drug as levels of MTX-polyglutamates correlate with clinical response in patients with RA [56].

## 4. MTX Therapeutic Mechanisms of Action in Inflammatory Settings

### 4.1. Folate Antagonism

MTX is a drug that has seen wide applications in the treatment of malignant diseases. Its effectiveness is attributed to its ability to inhibit key enzymes involved in the biosynthesis of purines and pyrimidines, thereby limiting malignant cell turnover [2]. As a potent inhibitor of dihydrofolate reductase (Figure 2), it reduces metabolically active intracellular folates decreasing the de novo synthesis of purines and pyrimidines (precursors of DNA and RNA) required for cellular proliferation [2]. It is, therefore, not surprising that it may find application in inflammatory diseases with a high turnover of inflammatory cells by inhibiting proliferation of the most rapidly dividing lymphocytes or other cells responsible for inflammation [1]. MTX-mediated effects on T cell proliferation either in vitro or in vivo have been demonstrated. MTX at low concentrations was shown to inhibit purine synthesis and hence ATP and GTP pools in primary human T lymphocytes. Low doses MTX were found to be cytostatic and not cytotoxic, halting proliferation of mitogen stimulated T cells [57]. In another study, Genestier and colleagues reported that low-dose MTX diminished antigen-dependent proliferation of T cells, taken from RA patients, through the induction of apoptosis. The effects of MTX on T cell function were completely reversed by thymidine or folinic acid addition [58]. Clonal growth of T and B cells obtained from both peripheral blood and rheumatoid synovial tissues, but not synovial adherent cells, was inhibited by clinically relevant concentrations of MTX. Hypoxanthine and thymidine supplementation reversed MTX suppressive effects on T cell growth. Adherent synovial cells resistance to MTX was suggested to be associated with slow proliferation and the ability to synthesize nucleotides via salvage pathways [59]. 

Despite the in vitro and in vivo data, the observation that folate supplementation is not associated with any reduction in anti-inflammatory efficacy of MTX in patients with RA is strong evidence that other mechanisms must account for the anti-inflammatory effects of the drug [15,60].

### 4.2. Effects on Extracellular Adenosine Accumulation

Adenosine is a potent anti-inflammatory mediator acting via interactions with a variety of immune-cell subtypes including neutrophils, macrophages, and T cells and modulating a vast array of physiological functions [61]. Several in vitro and in vivo studies provided evidence that enhanced adenosine levels mediate the anti-inflammatory effects of MTX as described below. 

Adenosine acts via four G-protein-coupled 7-transmembrene-receptors (ADOR); adenosine receptor A1 (ADORA1), adenosine receptor A2a (ADORA2A), adenosine receptor A2b (ADORA2B), and adenosine receptor A3 (ADORA3). Adenosine anti-inflammatory effects are largely thought to be mediated via ADORA2A, and possibly ADORA3 stimulation [62,63,64]. Activation of the ADORA2A and ADORA3 results in a reduction of pro-inflammatory cytokines IL-1β, TNF-α, and IL-6, and reduced matrix metalloproteinases (MMPs) production [65,66].

The role of adenosine as a mediator of MTX anti-inflammatory activity has been proposed since the observation of the inhibitory effect of polyglutamated MTX on 5-aminoimidazole-4-carboxamide ribonucleotide (AICAR) formyltransferase (ATIC), an enzyme required for de novo purine synthesis [53,67].

Inhibition of ATIC by MTX results in intracellular accumulation of AICAR and its metabolites, which are inhibitors of two key enzymes involved in the catabolism of adenosine and adenosine monophosphate (AMP): adenosine deaminase (ADA) and AMP deaminase (AMPD) [67]. By reducing the catabolism of adenosine and AMP, extracellular adenosine levels increase both directly and indirectly (by the dephosphorylation of increased adenine nucleotides). Of note, the generation of adenosine seems to be largely associated to the increased ATP and ADP release and cleavage (dephosphorylation) by the serial actions of the cell surface ectoenzymes ectonucleoside tri phosphate diphosphohydrolase 1 (also known as CD39) and ecto-5’- nucleotidase (also known as CD73), rather than direct adenosine release [53,68]. CD39 cleaves ATP and ADP to AMP, and CD73 cleaves AMP to release adenosine [62].

In vitro, MTX treatment increased adenosine release from cultured human fibroblasts and endothelial cells and the released adenosine reduced neutrophils adhesion to fibroblasts. This effect was reversed by the addition of ADA [69].

In the murine air pouch model of inflammation, weekly intraperitoneal injection of low-dose MTX increased adenosine concentration in inflammatory exudates and inhibited leukocyte accumulation at an inflamed site. Injection of ADA or an ADORA2A antagonist but not an ADORA1 antagonist reversed the effect of MTX on leukocyte accumulation [70].

The role of adenosine as a mediator of the anti-inflammatory effect of MTX has also been further investigated in gene-knockout mice models. In a murine air-pouch model of acute inflammation, MTX treatment increased the adenosine levels in the exudates of all strains of mice. In wild type mice, but not in ADORA2A and ADORA3-knockout mice, MTX reduced leukocyte and TNF-α accumulation in inflammatory exudates, suggesting that both adenosine A2A and A3 receptors contribute to the anti-inflammatory effects of MTX treatment in the air pouch model of inflammation [71].

In a mouse model of peritonitis, MTX increased adenosine concentration in peritoneal exudates of all mice studied, and reduced the leukocyte accumulation and TNF-α levels in the wild-type mice and ADORA3 knockout mice but not in ADORA2A knockout mice suggesting that ADORA2A is the principal anti-inflammatory adenosine receptor in this model [72]. 

These mechanisms of actions of MTX have been deciphered also in a 5′-nucleosidase (CD73)-deficient murine model associated with enhanced levels of TNF-α. MTX treatment reduced TNF levels in the exudates and increased exudate adenosine concentrations in wild-type mice, but not in CD73-deficient mice, suggesting that the majority of extracellular adenosine is derived from the cleavage of released adenine nucleotides [68].

MTX has also been shown to induce adenosine release in humans. Riksen and colleagues demonstrated that MTX inhibits ADA in lymphocytes and potentiates adenosine-induced vasodilatation [73]. Despite the difficulties to quantify MTX induced adenosine release, owing to the extremely short half-life of adenosine in blood and tissues, the effects on adenosine antagonism by caffeine has been studied [1]. Caffeine is a non-selective adenosine receptor antagonist which has been proven to reverse the beneficial effects of MTX treatment in a rat adjuvant arthritis model [74]. However, the results of adenosine receptor blockade by caffeine on treatment outcome of MTX treated patients are conflicting. A study by Nesher et al. showed that high caffeine intakes are associated with reduced efficacy of MTX [75], while Benito-Garcia et al found no significant effect of this agent on the effectiveness of MTX [76].

### 4.3. Effects on Polyamine Production

The ability of MTX to antagonize DHFR gives rise to decreased production of tetrahydrofolate (THF) and methyltetrahydrofolate (methyl THF) (Figure 2) which are methyl donors in chemical reactions. THF and methyl THF are involved in the production of methionine and S-Adenosylmethionine (SAM) and ultimately polyamines [2]. Polyamines, including spermine and spermidine, were found to accumulate in urine, synovial fluid and synovial tissue from patients with RA [77]. These polyamines are in turn, converted by monocytes into lymphotoxic products, such as ammonia and hydrogen peroxide [1]. By inhibiting DHFR, MTX decreases downstream mediators including methionine and SAM, thus decreasing methylation and subsequent formation of the potentially toxic transmethylation products, spermine and spermidine and therefore lymphotoxins [78]. However, other results suggested that the downregulation of polyamines synthesis does not explain the efficacy of MTX in RA. The transmethylation inhibitor, 3-deazaadenosine, showed antagonism of transmethylation in vivo, but had no clinical effects in RA [2,13,79]. Polyamine inhibition may therefore contribute to MTX efficacy, but does not seem to be the major mechanism of action of MTX.

### 4.4. Generation of Reactive Oxygen Species

MTX redox-altering properties have been proposed as an important mechanism of the immunosuppressive effects of the drug [80,81]. Low-dose MTX was found to induce transformed T cells apoptosis through the generation of reactive oxygen species (ROS) [80,81]. ROS are involved not only in cell death, but also modulate different cell functions, such as suppression of cytokine production and cell proliferation [80].

MTX-induced ROS generation may be mediated by tetrahydrobiopterin depletion. MTX is a potent inhibitor of DHFR. DHFR catalyzes reduction of dihydrobiopterin to tetrahydrobiopterin which is a required cofactor for NO synthesis by nitric oxide synthases (NOSs) [82]. Depletion of tetrahydrobiopterin uncouples NOS, leading to loss of NO synthesis and an increase in ROS production [82]. 

In mice, both tetrahydrobiopterin and dihydrobiopterin bind endothelial NOS (eNOS) with equal affinity. Tetrahydrobiopterin drives NO production, whereas dihydrobiopterin promotes eNOS uncoupling and superoxide production [83]. 

MTX treatment resulted in an elevation in dihydrobiopterin levels and a decreased tetrahydrobiopterin: dihydrobiopterin ratio in wild-type mice. These MTX-induced effects were magnified in tetrahydrobiopterin-deficient mice with marked reduced eNOS activity and increased superoxide generation [83].

In vitro, MTX-induced apoptosis in transformed human T cell line (jurkat cells) was found to be mediated by increased production of ROS and Jun-N-terminal kinase (JNK) activation. These MTX-induced effects were reversed by the addition of tetrahydrobiopterin [84].

In contrast to T cells, MTX failed to induce ROS synthesis and JNK activation in FLSs, apparently because of the extremely low levels of NOS enzymes in FLSs compared to T cells [84].

In another study, MTX was shown to inhibit IL6-induced ROS generation and cell proliferation [85].

### 4.5. Effects on Cytokine Production

Cytokines are major mediators in inflammatory and immune responses and have been of great interest in recent therapeutic developments in chronic arthritis, initially with TNF-α as a pivotal targeted cytokine [86].

The extent to which MTX modulates the pathogenesis of inflammatory autoimmune diseases via a direct effect on cytokine production by immune cells remains to be fully elucidated. 

**Figure 3:**
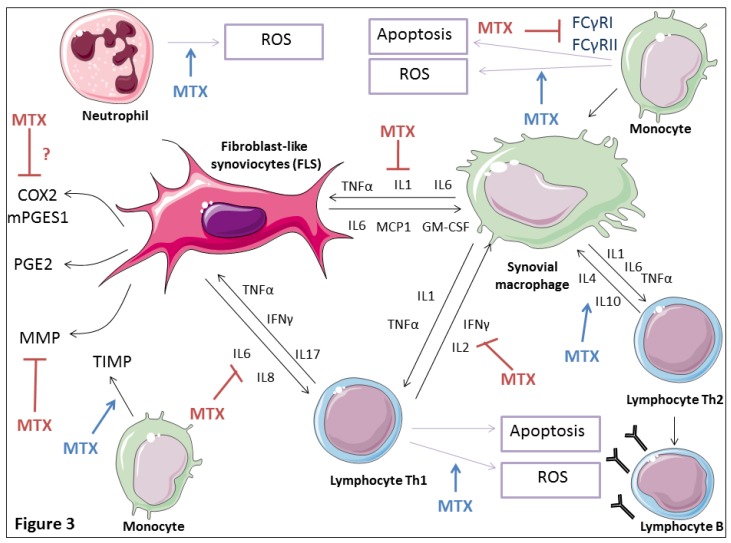
Immune regulatory action of low dose MTX in the RA synovial tissue. MTX treatment reduces proinflammatory monocytic/macrophagic cytokine (IL1β, IL6, and TNF-α) production, increases Th2 anti-inflammatory cytokine (IL4 and L10) gene expression, and decreases Th1 proinflammatory cytokine (IL2 and IFNγ) gene expression. MTX downregulates IgG Fc receptors FcγRI and IIa expression levels on monocytes decreasing their activation. MTX seems to disrupt synovial fibroblasts and T cells cross-talk signals by inducing inhibition of IL15, IL6, and IL8 expression by synovial fibroblasts, as well as IFNγ and IL17 expression in co-cultured RA T lymphocytes. MTX increases ROS synthesis in T cells, monocytes and neutrophils. MTX reduces T cells and monocytes growth and increases their apoptosis through the generation of ROS. MTX seems to have inhibitory effect on prostaglandin E2 (PGE2) production as well as on the expression of its synthesizing enzymes microsomal prostaglandin E2 synthase 1 (mPGES-1) and cyclooxygenase (COX) 2. MTX reduces synovial metalloproteinase (MMP) production and stimulates their inhibitors (TIMPs).

MTX has been reported to decrease TNF-α, IL1β, and adhesion molecules (E-selectin and VCAM-1) expression on RA synovial biopsies [87] (Figure 3). 

MTX was also suggested to be able to interfere with the binding of IL1β to its receptor without affecting the integrity of the IL1 receptor or of the target cells, and therefore inhibit the cellular response to IL1β [88]. In a murine collagen-induced arthritis model, intraperitoneal MTX injection reduced TNF-α serum levels and production by splenic T cells and macrophages [89]. MTX strongly suppressed TNF production by T cells receptor primed T lymphocytes from both healthy donors and RA patients [90]. MTX treatment decreased the proportion of TNF-positive CD4+ T cells in peripheral blood of patients with RA, and increased the number of IL-10-positive T cells [91]. Furthermore, MTX reduced the production of TNF, granulocyte macrophage colony-stimulating factor (GM-CSF), IFNγ, and IL-13 in T cells isolated from patients with RA. This suppression of cytokines was suggested to be due to the inhibition of the de novo synthesis of purines and pyrimidines since the addition of folinic acid or thymidine and hypoxanthine reversed the inhibitory effects of MTX on cytokine production [92]. MTX was also suggested to suppress TNF-α activity by suppressing NF-κB activation in vitro [84].

Co-culture of synovial fibroblasts and T cells from RA patients induced T cell IL-17, TNF-α, and IFNγ expression that contributed to increase fibroblast IL-15, IL-6, and IL8 expression. MTX inhibited upregulation of IL-15, IL-6, and IL8 by RA synovial fibroblasts stimulated by RA T cells. At the same time, MTX reduced IL-17 and IFNγ expression in T cells co-cultured with RA synovial fibroblasts (Figure 3). The effect of MTX on RA synovial fibroblasts/ T cells cross-talk signals was suggested to be mediated by adenosine release and decreased cell adhesion [93]. In vitro, MTX was shown to induce IL-4 and IL-10 gene expression, while decreasing that of IL-2 and IFNγ in peripheral blood mononuclear cells (PBMC) obtained from patients with RA [94]. Inhibition of IL6 secretion by cultured human monocytes may also represent a short-term anti-inflammatory effect of MTX [95].

MTX was also shown to suppress the IL-6 induced generation of ROS in the synoviocytes of RA patients [85]. Furthermore, MTX inhibited IL1 induced proliferation of T cells without affecting IL1 production or secretion [96]. MTX was also found to abolish the effects of PDGF and IL-1β and to inhibit mitogen-promoted FLS proliferation [97].

In RA synovitis, proinflammatory cytokines such as IL1β and TNF-α are mainly produced by monocytes and activated macrophages [98]. Activation of IgG Fc receptors by immune complexes such as rheumatoid factor (RF) may cause powerful activation of monocytes and macrophages. MTX was shown, both ex vivo and in vitro, to downregulate activating FcγRI and IIa on monocytes of RA patients [99] (Figure 3).

### 4.6. Effects on Matrix Metalloproteinases 

The MMPs are a family of Zn^2+^ dependent extracellular enzymes that play an important role in physiological and pathological tissue remodeling [100]. MMPs are synthesized as inactive proenzymes (pro-MMPs) and most of them are activated outside the cell by proteolytic cleavage [100]. Extracellulary, the activity of MMPs is regulated by their endogeneous inhibitors named tissue inhibitors of MMPs (TIMPs). Joint destruction in pathological conditions such as RA is probably due to an imbalance between activated MMPs and TIMPs in favor of MMPs [101].

In patients with RA, treatment with MTX downregulated serum levels of MMP-1, MMP-3, MMP-9, MMP-13, and TIMP-1 resulting on reduced ratios of MMP to TIMP [102]. Furthermore, systemic levels of activated MMPs were reduced in RA patients treated with MTX [103].

PBMC of RA patients treated with MTX exhibited enhanced production of TIMP-1 ex vivo. However, MMP-1 production in PBMC culture supernatants was not modulated by MTX [104]. 

MTX therapy decreased collagenase gene expression in synovial tissues from patients with RA, whereas TIMP-1 mRNA levels were unchanged [105]. In vitro, MTX did not alter collagenase or TIMP-1 mRNA levels in FLS exposed to IL1β [105].

IL-1 and TNF-α are potent inducers of MMP gene expression by FLS [106]. MTX inhibitory effect on MMP expression was suggested to be mediated by IL1β downregulation rather than a direct influence on MMP gene expression [95].

### 4.7. Effects on Prostaglandin Production

Prostaglandins (PGs) are major mediators of joint damage in RA. PGs synthesis is mediated by an enzyme cascade initiated by the release of arachidonic acid by a phospholipase [107]. Prostaglandin E2 (PGE2) is one of the predominant catabolic factors involved in RA. PGE2 acts as a mediator of pain and inflammation and promotes bone destruction [108]. High levels of PGE2 are detected in the synovial fluid of rheumatoid joints with strong expression in the synovium of its synthesizing enzymes as microsomal prostaglandin E2 synthase 1 (mPGES-1) as well as cyclooxygenase (COX) 1 and 2 [107,108].

Very limited data are available about the effects of MTX on PGE2 production. MTX was suggested to have inhibitory effect on PGE2 synthesis (Figure 3); Administered in experimentally induced RA rat model, MTX was shown to slow-down the rate of joint destruction by reducing COX2 joint-destructive enzyme [109]. In a rabbit model of antigen induced arthritis, MTX significantly reduced intra-articular levels of PGE2 compared to saline treated controls [110]. Added in vitro, MTX strongly reduced PGE2 release from rat peritoneal macrophages [111]. In cultured synovial cells from RA patients, MTX caused a dose-dependent decrease on IL-1 induced PGE2 production, without affecting COX1 and COX2 mRNA expression [112]. PGE2 production by COX2 was found to be reduced in whole blood from MTX-treated patients with RA in comparison with healthy controls. However, in vitro incubation of MTX with blood obtained from healthy donors, showed no direct MTX inhibitory effect on COX2 and COX1 activities, suggesting that MTX effects on serum PGE2 production may be mediated by a serum factor induced by MTX or by a MTX metabolite [113]. Changes on PGE2 production may also represent a downstream effect of the influence of MTX on IL1 production [53].

Other studies demonstrated that MTX failed to elicit a change in PGE2 synthesis. MTX had no effect on COX metabolic pathway in adjuvant arthritic rats [114]. MTX did not affect mPGES-1, COX-1, and COX-2 expressions in synovial tissue biopsies from RA patients [115]. Moreover, MTX had little effect on IL1-induced PGE2 production by RA synovial fibroblasts [116].

### 4.8. MTX Inhibits NF-κB Activity

**Figure 4:**
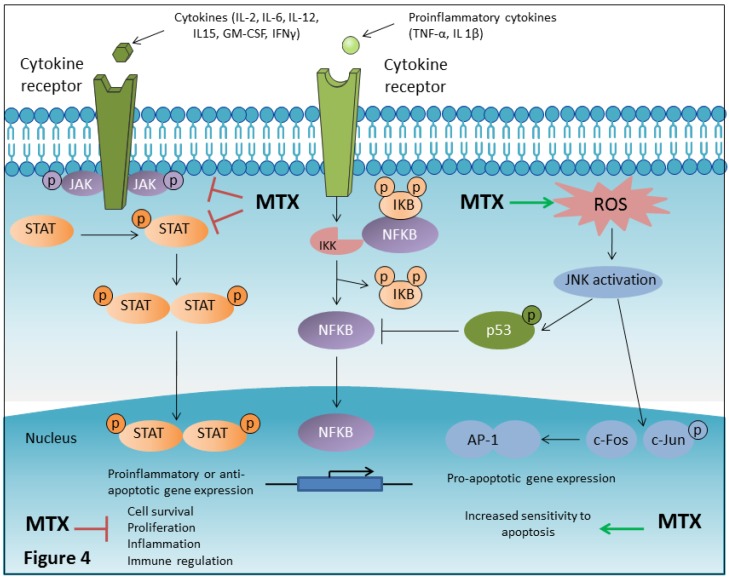
MTX affects several signal transduction pathways implicated in the pathogenesis of rheumatoid arthritis (RA). Dysfunctional intracellular signaling involving deregulated activation of the Janus Kinase/Signal Transducers and Activators of Transcription (JAK/STAT) and nuclear factor-κB (NF-κB) activation pathways play a critical role in RA. MTX seems to be a potent suppressor of JAK/STAT signaling pathway which is responsible for the transduction of multiple pro-inflammatory cytokines implicated in the pathogenesis of RA. MTX reduces JAK1, JAK2, STAT1, and STAT5 phosphorylation. Low dose MTX strongly suppresses levels of phosphorylated STAT5. Moreover, MTX-mediated ROS production activates the mitogen-activated protein kinase (MAPK), Jun-N-terminal kinase (JNK), and JNK-dependent induction of p53, which is the final mediator of inhibition of NF-κB activation. NF-κB is involved in inflammation, immune response and cell proliferation and survival. MTX-mediated JNK activation also activates prototypical JNK downstream targets, c-JUN and c-FOS, components of the AP-1 complex which is involved in the increased expression of pro apoptotic genes (such as TNF-α, Fas-L, and Bak) mediating increased sensitivity of cells to apoptosis.

Nuclear factor-κB (NF-κB) is a well-established cytoplasmic transcription factor involved in inflammation, immune response as well as in cell proliferation and apoptosis [117]. It is activated by a broad panel of different stimuli including pro-inflammatory cytokines like TNF-α or IL 1β [117,118]. In unstimulated cells, NF-κB resides in the cytoplasm in an inactive form associated with regulatory proteins called inhibitors of κB (IκB) [119]. Phosphorylation of IκB, which is mediated by the inhibitory kappa B kinase (IKK), is an important step in NF-κB activation. Upon phosphorylation, the IkB dissociates from NF-κB, allowing the ‘active’ NF-κB to translocate to the nucleus and activate the expression of NF-κB target genes (Figure 4) [119,120].

P53 is a transcription factor that inhibits cell proliferation and induces apoptosis [121]. RA is characterized by both elevated NF-κB activity and p53 deficiency [119,122,123].

RA subjects not receiving MTX were found to exhibit chronic activation of NF-κB in CD4+ T cells compared with controls and MTX normalized elevated NF-κB activity in RA patients [84].

LincRNA-p21, a long intergenic non-coding RNA, has been proposed as a negative regulator of NF-κB activity. MTX was found to inhibit TNF-α-induced NF-κB activity via lincRNA-p21 activation in cultured T cells [124].

In both Jurkat T cells and primary human T cells, MTX inhibited NF-κB activation through MTX-dependent BH4 depletion, and increased ROS production and activation of JNK and p53 [84] (Figure 4). Importantly, MTX also inhibited NF-κB activity in FLSs [84]. In contrast to T cells, inhibition of NF-κB activity in FLSs was found to be independent of p53 induction. MTX inhibitory effect on NF-κB activity was suggested to be mediated by adenosine release and activation of A2A and A3 adenosine receptors in FLSs [84].

MTX inhibits production of inflammatory mediators such as and IL-6, TNF-α, IL1β, and MMPs [85,87,102]. The transcription of these mediators is known to require activation of NF-κB [119]. MTX inhibitory effects on inflammatory responses may be therefore mediated by NF-κB signaling suppression.

### 4.9. MTX is a JAK/STAT Pathway Inhibitor

Activated by numerous cytokines and growth factors, JAK/STAT signaling pathway plays a critical role in inflammatory response as it contributes to the significant upregulation of pro-inflammatory cytokine expression as well as aberrant cell survival which are both associated with RA [125]. The canonical JAK/STAT pathway is responsible for the transduction of multiple pro-inflammatory cytokines implicated in the pathogenesis of RA, including IL-2, IL-6, IL-12, IL15, GM-CSF, and IFNγ [126,127]. The binding of these cytokines to their membrane receptors leads to the activation of associated Janus kinases (JAKs) through a process of auto- or transphosphorylation on cytokine-induced receptor dimerization. These active JAKs are then able to phosphorylate specific tyrosine residues on the cytoplasmic tails of the cytokine receptors creating binding sites for signal transducers and activators of transcription (STATs). STATs are then phosphorylated by JAKs converting them to active transcription factors that translocate to the nucleus and drive the expression of multiple genes that are important for cell activation, localization, survival, and proliferation [127].

Recently, MTX has been classified as an inhibitor of (JAK/STAT) signaling pathway [128] (Figure 4). MTX was found to induce a dose dependent reduction of constitutive JAK1 phosphorylation in the Hodgkin lymphoma cell line HDLM-2. Moreover, MTX produced a dose-responsive reduction of both STAT1 and STAT5 phosphorylation [128]. Low-dose MTX was shown to strongly reduce levels of tyrosine phosphorylated STAT5, without affecting other phosphorylation-dependent pathways (Akt, cJun, and ERK1/2) [128]. MTX inhibitory effect on JAK/STAT signaling was found to be independent of its effects on folate metabolism, as suppression of STAT phosphorylation was not reversed in the presence of folinic acid. The exact molecular mechanism through which MTX may control the JAK/STAT pathway remains unknown. However, the effect of MTX on the JAK/STAT pathway suppression may represent a principal anti-inflammatory and immunosuppressive mechanism of action of low-dose MTX. MTX may ‘dampen’ the pathological over-activation of the JAK/STAT pathway allowing the control of inflammatory diseases without preventing physiological activation [128,129].

### 4.10. MTX Inhibits Proinflammatory HMGBI Alarmin Effects

High-mobility group box chromosomal protein 1 (HMGB1), also called amphoterin or HMG1, is a 25 to 30-kDa abundant non-histone nuclear protein constitutively expressed in the nucleus of eukaryotic cells [130,131].

When released by activated immune cells (macrophages, monocytes, and dendritic cells) or by injured cells, HMGB1 acts as an important mediator of inflammation or alarmin and promotes acute inflammation and subsequent tissue repair [130,131,132]. HMGB1 functions are mediated, at least in part, by receptor for advanced glycation end-products (RAGE). RAGE is a member of the immunoglobulin super family of cell surface molecules. It is involved in the pathogenesis of a various inflammation associated diseases such as diabetic complications and chronic immune inflammatory disorders [133,134]. In addition to RAGE, TLR2/4, receptors for LPS, are also implicated as HMGB1 receptors [130].

Previous studies have shown that HMGB1 is associated with chronic autoimmune inflammatory diseases including RA [135]. HMGB1 was shown to be overexpressed in the synovial biopsy of rheumatoid and experimental arthritis [136,137]. Injection of recombinant HMGB1 (rHMGB1) into mice induced arthritis with mild to moderate synovitis and pannus formation [138,139]. Based on its association with the development of autoimmune inflammatory disorders, HMGB1 has received increasing attention in RA research.

**Figure 5:**
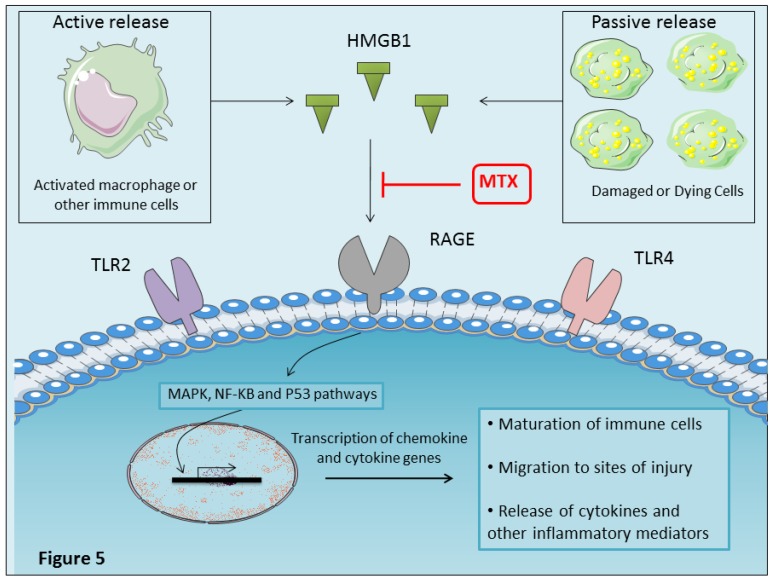
MTX inhibits HMGB-1/RAGE interaction. HMGB1 released by activated immune cells (macrophages, monocytes, and dendritic cells) or by injured cells acts as an important mediator of inflammation or alarmin. HMGB-1 activates cells through the engagement of multiple surface receptors including TLR2, TLR4, and RAGE. Downstream signaling of HMGB1 is mediated by a number of adaptor proteins, which converge through pathways involving mitogen-activated protein kinase (MAPK) and nuclear factor kappa B (NFκB) and transcriptional regulator, p53 pathways (Weber et al. 2015). HMGB-1 signaling through RAGE promotes maturation of immune cells, chemotaxis and release of pro-inflammatory cytokines (TNF-α, IL-1, IL-6, and IL-8). MTX can bind to the RAGE binding domains of HMGB1 and inhibits the interaction between HMGB1 and its receptor RAGE, thus inhibiting the development of inflammatory responses. Binding of MTX to part of the RAGE-binding region in HMGB1 may be significant for the anti-inflammatory effect of MTX.

MTX was found to reduce HMGB1 expression in RA synovial tissues and to inhibit disease progression [140]. A relevant study showed that biotinylated MTX can bind to the K86-V175 fragment of HMGB1, which is the RAGE binding domains, to inhibit the interaction between HMGB1 and its receptor RAGE, thus inhibiting the development of inflammatory responses [141] (Figure 5).

Mononuclear macrophages can actively release HMGB1 in response to TNF-α and IL1β [135]. MTX has been reported to downregulate TNF-α and IL1β expression on RA synovial biopsies [87]. The suppression of TNF-α and IL1β by MTX can lead to downregulation of HMGB1. MTX can therefore inhibit the HMGB1 inflammatory effect either directly by binding to RAGE-binding region in HMGB1 or indirectly by suppressing inflammatory cytokine production.

## 5. MTX Adverse Effects Mechanisms of Action

Despite its widespread use in various autoimmune and inflammatory disorders, low-dose MTX is not free of drug toxicity. The most common MTX-related adverse reactions are gastrointestinal manifestations (nausea, vomiting, stomatitis, loss of appetite) and hepatotoxicity [17]. Generally, the main cause of MTX treatment withdrawal is not the lack of efficacy but toxicity [17]. Careful and appropriate patient monitoring (blood cell counts, hepatic enzymes, creatinine) should be performed periodically and appears to significantly minimize risks associated with MTX use [142].

The mechanisms of MTX toxicity remain unclear. Some toxicities—such as cytopenia, gastrointestinal intolerance, and stomatitis—mimic the manifestations of folate deficiency and can be prevented or alleviated by folic or folinic acid supplementation [25]. Toxicities unrelated to folate deficiency include nodulosis, pulmonary fibrosis, lethargy, fatigue, and renal insufficiency [14]. The understanding of the molecular mechanisms of action of MTX may help to explain many of MTX associated toxicities [14] (Table 1).

One of the major adverse effects of MTX is hepatotoxicity. Minor elevations in aminotransferases are common, but hepatic steatosis, fibrosis, and cirrhosis occur infrequently during low-dose MTX therapy [8]. The mechanism by which MTX adversely affects the liver remains unclear. It was suggested that MTX hepatotoxicity may result from a depletion of hepatic folate stores and the accumulation of MTX poly glutamates in the liver [150]. A definitive relationship between folate deficiency and hepatotoxicity has not been experimentally confirmed. However, folate supplementation has been associated with a reduced incidence of hepatic adverse effects (elevated transaminases) induced by MTX treatment [8,15].

MTX-related hepatic fibrosis was found to be mediated through an adenosine pathway. MTX was shown to enhance the release of adenosine from cultured hepatoma cells [151]. Ethanol, which is one of the most important causes of hepatic cirrhosis, has the same effect on hepatocyte release of adenosine [190]. Adenosine, in turn, binds to the adenosine A2A receptor on hepatic stellate cells, the principal fibrogenic cell type in the liver, and promotes collagen production [151,152]. Unlike wild-type mice, mice deficient for the adenosine A2A receptor were protected from developing liver fibrosis when challenged by hepatotoxin (carbon tetrachloride or thiocetamide) demonstrating the key role of adenosine A2A receptors in the pathogenesis of hepatic fibrosis [151].

Moreover, MTX is known to interfere with the generation of methionine from homocysteine (Figure 2). Excess homocysteine can induce endoplasmic reticulum stress and promote fat accumulation in the liver. Homocysteine can also activate proinflammatory cytokines and hepatic stellate cells, leading to liver fibrosis [153,154].

MTX demonstrates important toxic effects on the pulmonary system. The pathogenesis of MTX associated pulmonary toxicity has not been elucidated fully.

Acute pneumonitis is the most common type of pulmonary toxicity associated with MTX. Most researchers suggest that MTX pneumonitis is a form of hypersensitivity lung disease because of the presence of fever, peripheral eosinophilia, an increase in CD4+ (T-helper) cells in bronchoalveolar lavage fluid, as well as a mononuclear cell infiltration of the lungs and granulomatous inflammation [157,158,159]. However, others suggest that injury may result from a direct toxic effect of MTX on the lung [191]. Evidence for MTX direct pulmonary toxicity has been proposed by Ohbayashi and colleagues; MTX was shown to induce alveolar epithelial injury and pulmonary fibrosis with a decrease of alveolar epithelial cells and an increase of fibroblast cells in mouse lung tissues [160]. Other researchers speculate that the immunosuppressive effects of MTX impair the host immune response and increase the susceptibility of the patient to acquired or latent viral infections (e.g., cytomegalovirus or Epstein–Barr virus) [161]. Moreover, MTX was found to induce MAPK pathways activation and to modulate cytokine expression which may contribute to the pulmonary inflammatory response [156].

Interestingly, MTX was reported to be associated with marked asthenia in patients [171]. This is possibly due to the release of adenosine in the CNS. Adenosine is known to have neuromodulatory properties and its accumulation in the CNS is associated with headache, nausea, and somnolence [192]. By acting at the A1 receptor on the perifornical lateral hypothalamus, adenosine may regulate wakefulness and somnolence, so potentially explaining the sleepiness experienced by some patients after MTX intake [171]. In children receiving high doses of MTX, severe sleepiness and coma have been described [172]. Bernini and colleagues reported that theophylline, a non-selective adenosine receptor antagonist, could reverse the CNS toxicity of MTX in children treated with high doses of MTX [172].

MTX-induced neurotoxicity may also be mediated by elevated homocysteine levels and their excitatory amino acid neurotransmitter metabolites, such as homocysteic acid and cysteine sulfinic acid, which may cause excitotoxic neural death [173].

An impairment of biopterin metabolism, leading to decreased monoamine neurotransmitters synthesis was also suggested as a possible mechanism of MTX associated neurotoxicity [174].

MTX treatment is known to induce the formation of subcutaneous nodules, an accumulation of multinucleated giant cells derived from mononuclear cells [169,170,193]. Using an in vitro model of giant cell formation, Merrill and colleagues investigated MTX-induced nodulosis. They demonstrated that MTX enhances the generation of multinucleated giant cells, as does adenosine A1 receptor occupancy. This effect of MTX is reversed by a specific adenosine A1 receptor antagonist. Thus, MTX-induced nodulosis may be mediated by adenosine through the adenosine A1 receptor [169].

It is well-documented that low-dose MTX can exhibit kidney damage [162,163,164,194]. However, the mechanism underlying MTX-induced kidney injury remains unknown. It has been reported that high dose MTX can cause kidney damage by the precipitation of MTX and its major metabolite, 7-OH MTX, in acid urine which may contribute to intratubular obstruction and impaired renal function [37,49,165]. Kidney damage due to precipitation of MTX and tubular injury may occur with high dose MTX; but it is very rare with chronic low dose therapy. It was also suggested that MTX-associated renal injury may by mediated through the induction of adenosine plasma concentration and subsequent activation of A1 receptors in renal parenchyma, reducing renal blood flow and thereby diminishing renal function [167]. Recently, using a rat model with renal failure caused by low-dose MTX administration, Li et al. demonstrated that long-MTX administration caused MTX accumulation in renal tissue and severe glomerular and tubular injury through an increase in oxidative stress [168].

MTX is largely excreted into urine. Impaired renal excretion of MTX and its accumulation in serum may lead to the enhancement of MTX toxicities and primarily bone marrow depression [195]. For this reason, low dose MTX is contraindicated if the glomerular filtration rate (GFR) is less than 30 mL/min [196].

MTX should also not be delivered to pregnant women, due to risks of fetal death or malformations [49]. Some cases of MTX-induced lymphomas have also been reported, potentially related to EBV. MTX may also induce severe skin reactions and opportunistic infections such as *Pneumocystis carinii* pneumonia [25,159].

## 6. MTX Response Variability

There is a considerable inter-individual heterogeneity in clinical response to MTX, both in terms of efficacy and toxicity, with response varying from 50–70% as defined by the American College of Rheumatology (ACR 20) criteria [28,197,198].The inter-patient variability in MTX effects is related to various contributing factors, including individual patient factors (age, sex, ethnicity, co-morbidities), disease specific factors (disease duration, severity, activity) and genetic factors [199]. Specifically, polymorphisms in genes coding for MTX transport and metabolism (SLC19A1/RFC, the solute carrier organic anion transporter 1B1 (SLCO1B1), FPGS, GGH, and ABCB1), for folate pathway genes (MTHFR, DHFR, TYMS) and polymorphisms in adenosine pathway genes (ATIC, AMPD1, ADA, inosine triphosphate pyrophosphatase (ITPA), (MS/MTR) and MTRR) demonstrate association with the MTX response [198].

A recent systematic review reported associations between MTX response in RA patients and single-nucleotide polymorphisms (SNPs) in the MTHFR gene 1298A>C (rs1801131), ATIC gene 347C>G (rs2372536), RFC-1 gene 80G>A (rs1051266), SLC19A1 A>G (rs2838956) and SLC19A1 gene G>A (rs7499) [200].

SNPs in the ATIC gene (rs12995526, rs3821353, rs7563206 and rs16853834), in the SLC19A1 gene region (rs11702425, rs2838956, rs7499, rs2274808, rs9977268 and rs7279445) and within the GGH gene (rs12681874) were associated with MTX efficacy. Other SNPs were significantly associated with adverse events; SNPs in the DHFR gene (rs12517451, rs10072026, and rs1643657) and in the FPGS gene [199]. A relationship between genetic variants in the adenosine biosynthesis pathway and outcomes of MTX treatment in patients with RA and JIA was also reported; polymorphisms in the AMPD1, ATIC, and ITPA genes were associated with good clinical response to MTX treatment [201,202,203].

ITPA enzyme catalyzes the conversion of iosine triphosphate (ITP) to iosine monophosphate (IMP) in the purine synthesis pathway. Deficiency of ITPA was reported to possibly influence its balance with AMP and adenosine [202]. Pastore et al. showed that reduced activity of ITPA is related to reduced MTX efficacy in patients with JIA [204].

Genome-wide association studies (GWAS) in patients with RA and JIA were carried out to analyze response to MTX therapy. Senapati et al. [205] identified potential risk loci for poor MTX response, including associations with the previously identified DHFR, FPGS, and TYMS genes. Cobb et al. [206] identified novel genes associated with MTX response in JIA patients including genes related to TGF beta signaling (*ZMIZ1*: zinc finger MIZ-type containing 1, *TGIF1*: TGFB-induced factor homeobox 1) and a member of the multi-drug resistance subfamily of the ATP-binding cassette transporter proteins (*CFTR:* cystic fibrosis transmembrane conductance regulator). A recent GWAS of response to MTX in 1424 early RA patients of European ancestry, reported a strong evidence for association of Neuregulin 3 (*NRG3*) gene with MTX response and supported the previously described association with ZMIZ1 gene [207].

## 7. MTX and Chronic Viral Arthritis

Many viruses have been associated with inflammatory arthralgias and arthritis. Old world alphaviruses, such as Chikungunya virus (CHIKV) and Ross river virus (RRV), Parvovirus B19, HBV/HBC, and HIV are among the most important causes of virally mediated arthritis [5]. Viral infections may manifest as acute or chronic arthritis. Most cases of viral arthritis are short-term and self-limited due to effective elimination of the pathogen by the immune system. Chronic arthropathies are associated with persistent or latent viral infections and/or virus induced autoimmunity [6]. In chronic viral arthritis, the joint manifestations can mimic those of RA and can endure for months to years [6].

Alphaviral arthritis involves infection of periosteal osteoblasts with secretion of proinflammatory cytokines (IL-1β, IL-6, and chemokines (CCL2). The recruitment of monocytes to joint sites of infection leads to emergence of osteoclast-like cells responsible for bone erosion and subsequently to arthritis [208,209].

Post-viral arthritis is mainly managed with analgesics and non-steroidal anti-inflammatory drugs (NSAIDs), which provide symptomatic relief but do not significantly, affect the underlying disease process [7].

Given pathogenic similarities with RA and the disabling nature of the viral induced arthritis, it is not surprising that DMARDs such as MTX have begun to be used in the treatment of viral arthritis. MTX as a safe, effective and widely used drug for the treatment of inflammatory rheumatic diseases, prompted a growing interest in the treatment of viral induced arthritis [4].

Chikungunya (CHIK) is a rapidly emerging viral infection that can cause chronic, debilitating inflammatory arthritis [210]. Chronic CHIKV-induced arthritis can cause joint damage and worsening quality of life as severe as RA. Therefore, there has been increased interest in the use of DMARDS, and particularly MTX, in the treatment of CHIK arthritis [4,211,212].

Several studies support the use of MTX in chronic CHIK arthritis. MTX at 15 mg/week led to a positive therapeutic response in 75% of the 72 patients with post CHIK chronic inflammatory rheumatisms (pCHIK-CIR) who met criteria for RA, spondyloarthropathy, or undifferentiated polyarthritis [213]. Addition of MTX to sulfasalazine and hydroxychloroquine in non-responder patients with chronic persistent CHIK arthritis, resulted in significant improvement of clinical responses (MTX vs no MTX, 71.4% vs. 12.5%, respectively) [214].

In a recent unblinded randomized study, Ravindran and Alias demonstrated the efficacy and superiority of MTX triple therapy (MTX 15mg/week, hydroxychloroquine 400 mg/day, and sulfasalazine 1 g/day) compared with hydroxychloroquine alone [215].

Several other studies have reported complete resolution of chronic joint symptoms after MTX treatment without significant toxicity [216,217,218].

Malvy et al. studied the cytokine profile before and 4 months after treatment with MTX in a patient presenting progressive erosive arthritis after CHIKV infection. Clinical and radiological improvement was observed after 4 months of MTX treatment. High levels of proinflammatory cytokines (IL-1β, IL-6, IL-8, IL-10, TNF-α, and IFNγ) were detected in lymphocyte supernatants prior to MTX treatment. These proinflammatory mediators were markedly decreased after MTX treatment [219].

MTX may therefore provide benefits in the treatment of chronic CHIKV induced arthritis. However, concerns are raised about the safety of MTX use in virally induced arthritis owing to the potential capacity of MTX, as an immunosuppressant drug, to promote viral replication. MTX was found to enhance disease onset and severity in RRV infected mice with a significant increase in viral load in sera and quadriceps in comparison to non-MTX-treated mice [220]. However, other in vitro and in vivo data showed that MTX did not affect alphavirus replication. In human synovial fibroblasts infected with different MOIs of CHIKV and particularly with very low MOI in order to mimic in situ tissue settings of chronically infected patients, MTX was found to have no effect on viral replication [221]. In a mouse model of CHIKV infection, MTX treatment did not increase viral load in target tissues when compared to non-treated mice [222].

Polyarthralgias and polyarthritis have been associated with HCV infection and are amongst the most frequent extrahepatic manifestations in chronically infected patients with HCV [5,223]. The treatment of HCV-related arthritis is poorly standardized and often empirical. NSAIDs, low doses of corticosteroids and hydroxychloroquine are used in the treatment of HCV chronic arthritis, whereas MTX has been used less frequently [223]. The clinical use of MTX in the context of HCV chronic arthritis has only been studied on a case-by-case basis, due to its well-known hepatic complications and immunosuppressive effect. Consequently, there is a paucity of studies demonstrating the efficacy as well as the safety of MTX in the treatment of arthritis associated with HCV [223].

In a retrospective study on a group of HCV patients with chronic inflammatory arthritis, Nissen et al. found MTX efficacious and well tolerated [224]. The safety of MTX therapy was also investigated in RA patients with chronic viral hepatitis HCV. In a study on 600 RA patients tested for HCV, the two patients who had been treated with MTX showed normal transaminase levels [225]. Moreover, treatment of hepatitis C-positive RA patients with MTX for up to 1 year did not result in liver cirrhosis [226].

Arthritis occurs during both the acute and chronic HBV infection [5]. HBcAg was found in the synovium of patients with RA with chronic HBV infection [227]. The presence of HBV in RA synovium was suggested to be involved in the pathogenesis of RA [227]. Some cases of HBV reactivation after MTX therapy in HBsAg-positive patients have been reported in the rheumatology literature mainly in the form of case reports [228,229].

More recent studies suggest that long-term MTX does not result in hepatitis B reactivation or on accelerated liver disease. Laohapand et al. found that none of HBV exposed patients had hepatitis B reactivation during an average of 9.9 years after MTX treatment [230]. In a population-based cohort study, Tang et al. reported no significant increase of liver cirrhosis in RA patients with chronic hepatitis C or B who received long-term MTX treatment [231,232]

The ACR guidelines recommend screening for hepatitis C and B in patients with risk factors prior to initiating MTX. Chronic hepatitis C or B is a contraindication for MTX therapy [196].

Various rheumatic manifestations with arthralgic disorders are commonly observed in HIV patients [233,234]. HIV-associated rheumatic syndromes can be managed with NSAIDs, DMARDs (e.g., MTX), and anti TNF-α therapies [234].

There are limited data on the use of MTX therapy for the treatment of rheumatic disease in HIV- positive patients [235].

Early reports of MTX treatment for HIV associated psoriatic disease discouraged its use because of the development of opportunistic infections (*Pneumocystis carinii* pneumonia and Staphylococcus sepsis) and toxicities (leukopenia and toxic encephalopathy) [236,237]. Other reports have suggested a potential beneficial effect of MTX therapy in HIV infected patients [238,239]. Maurer et al. reported a case series of three patients with psoriasis and psoriatic arthritis treated with MTX. Psoriasis and psoriatic arthritis improved in all patients. No opportunistic infections developed in two patients; one patient receiving chemotherapeutic levels MTX for treatment of concomitant AIDS-associated Kaposi sarcoma, developed *Pneumocystis carinii* pneumonia [238].

The management of rheumatic syndromes in the HIV-positive population is challenging. The decision to use low doses of MTX in cases of severe refractory disease should be made carefully with appropriate monitoring of HIV load and CD4+ counts. Concomitant prophylaxis for opportunistic infections and antiretroviral therapy should also be strongly considered [237].

## 8. Conclusions and Perspectives

A number of pharmacological mechanisms of low-dose MTX action have been proposed; MTX may induce inhibition of purine and pyrimidine synthesis, suppress transmethylation reactions and polyamines accumulation, and promote adenosine release with adenosine mediated suppression of inflammation and suppression of HMGB1 alarmin function. Some other proposed mechanisms of MTX activity include generation of ROS and alteration of MMPs production, PGE2 synthesis, and cytokine expression which may represent indirect effects rather than primary responses. Evidence also exists for MTX effects on several transduction pathways implicated in the pathogenesis of RA including JAK/STAT and NF-κB activation pathways. The disease modifying action of MTX is likely to result from a combination of all these mechanisms with pleiotropic therapeutic effects on various immune cells leading to an overall dampening of the inflammatory response. Low-dose MTX benefits a large number of patients suffering from various inflammatory autoimmune diseases but toxicity still a concern. Common side effects are observed in patients treated with MTX involving toxicities in a number of organs such as gastrointestinal tract and liver. MTX-related adverse effects may involve immunologic, inflammatory, or lymphoproliferative disorders. Improving the understanding of basic mechanisms of action of MTX provided further insights into MTX-associated toxicity. Some MTX-associated side effects are related to MTX mechanism of action and may be mediated through folate antagonism, enhanced adenosine release, or elevated homocysteine levels.

The understanding of the therapeutic and toxic mechanisms of action of MTX may be of further interest to identify new therapeutic targets to manage inflammatory and immune-mediated pathologies while minimizing the unwanted toxic effects. This is important for new drug design and the development of more targeted therapies to maintain efficacy while diminishing toxicity of the drug. MTX has also prompted a growing interest in the treatment of viral mediated arthritis due to its beneficial anti-inflammatory activities. The management of chronic viral-associated rheumatic syndromes with DMARDS is challenging and should provide favorable safety and efficacy profiles. The most apparent risk with the use of MTX in chronic viral arthritis is that of decreased immune surveillance in patients, creating a potential risk for viral reactivation in a context of viral persistence. Conflicting results have been reported about the tolerance of MTX in chronic viral mediated arthritis. Until now, the treatment of viral arthritis is still poorly standardized and often empirical. Official guidelines for clinicians are necessary. Further studies are highly warranted to evaluate the efficacy as well as the safety of MTX use in the management of viral-related rheumatic syndromes.

## Figures and Tables

**Table 1:** Major low dose MTX related adverse events.

Organ System	MTX Related Adverse Events	Toxic Mechanism of Action
Gastrointestinal	Nausea; Vomiting; Diarrhea; Mucositis and stomatitis	Gastrointestinal toxicities and bone marrow suppression seem to be directly related to folate antagonism, because these tissues have high cell turnover with a high requirement for purines, thymidine, and pyrimidine [37,143,144]. Supplementation of folic or folinic acid may diminish toxicity. Gastrointestinal symptoms of nausea and diarrhea may be more frequent with oral MTX [145]. Switching from oral to parental administration was shown to significantly decrease the frequency of adverse gastrointestinal events in patients with RA [146,147], suggesting that other mechanisms may account for MTX induced gastrointestinal toxicity. The pathogenic mechanism underlying gastrointestinal side effects may also be related to the change of plasma homocysteine [148].
Hematological	Anaemia; Leucopenia; Thrombopenia; Pancytopenia	Recently, MTX-induced thrombocytopenia was shown to be mediated by MTX-induced activation of platelet apoptosis via JNK and oxidative stress [149].
Hepatic	Elevated liver enzymes	Long-term MTX administration can cause accumulation of MTX polyglutamates in the liver and decreased folate levels. The depletion of hepatic folate stores by accumulated MTX poly glutamates is one possible toxic effect of MTX on the liver [150]. Folate supplementation has been associated with a reduced incidence of elevated transaminases induced by MTX treatment [15].
	Steatosis, fibrosis, cirrhosis	MTX-related hepatic fibrosis may be mediated through an adenosine pathway. MTX was shown to enhance adenosine release from cultured hepatoma (HepG2) cells. Adenosine A2A receptor occupancy stimulates collagen production by hepatic stellate cell lines [151,152]. Unlike wild-type mice, mice deficient for the adenosine A2A receptor or treated with an adenosine A2A receptor antagonist (ZM241385) were protected from developing liver fibrosis when challenged by hepatotoxin (carbon tetrachloride or thiocetamide) [151]. MTX-related liver fibrosis may also be mediated by its capacity to interfere with the generation of methionine from homocysteine. Excess of homocysteine induces endoplasmic reticulum stress promoting fat accumulation in the liver. Homocysteine can also activate hepatic stellate cells and proinflammatory cytokines, leading to liver fibrosis [153,154]. MTX-induced hepatic damage may be related to the generation of reactive oxygen species (ROS). MTX was shown to cause oxidative tissue damage by increasing lipid peroxidation in the liver tissue and decreasing the level of antioxidant enzymes in rats [155].
Pulmonary	Interstitial pneumonitis; *Pneumocystis carinii* pneumonia; Pulmonary fibrosis	Pulmonary toxicity has been shown to occur at both high- and low-dose MTX treatment, suggesting an idiosyncratic reaction not linked to folate antagonism [49]. Several mechanisms for the pathogenesis of MTX-induced pneumonitis have been suggested including hypersensitivity, direct drug toxicity to the lung tissue, immunosuppression or altered cytokine expression contributing to the pulmonary inflammatory response and tissue damage [156]. Typical bronchoalveolar lavage and histological findings support the concept that MTX-induced pneumonitis represents a hypersensitivity reaction [157,158,159]. MTX also induces injury to alveolar epithelial walls and pulmonary fibrosis, suggesting a direct drug toxicity route [160]. MTX pulmonary toxicity may be mediated by mitogen-activated protein kinase (MAPK) pathways activation and cytokine release [156]. MTX can compromise the immune response and increase the risk for opportunistic infections due to *Pneumocystis carinii* [161].
Renal	A decrease in glomerular filtration rate; Renal insufficiency (only in pre-existing, severely impaired renal function)	In contrast to high-dose MTX, which can lead to direct tubulus toxicity and subsequent renal failure, renal toxicities induced by low-dose MTX are rare. Low dose MTX has been associated with decrease in glomerular filtration rate (GFR) [162,163,164]. MTX and its major metabolite 7-OH-MTX are relatively insoluble in acid urine and may act as a direct toxin on the tubular epithelium, or precipitate within the tubular lumen, which can lead to intratubular obstruction resulting in a decrease in GFR (particularly at high doses) [49,165]. Evidence for a direct toxic effect of MTX on renal tubular cells has been demonstrated [166]; Low doses MTX can induce cell swelling and necrosis in renal tubular cells, which may lead to permanent tubular damage [166]. MTX associated renal toxicity may be explained by an increase in plasma adenosine concentration in extracellular fluid and subsequent activation of A1 receptors in renal tissue, reducing renal blood flow and salt and water excretion [167]. Long duration of low dose MTX administration caused severe kidney injury and renal MTX accumulation in a rat model. 4-hydroxynonenal (4-HNE) and malondialdehyde (MDA), which are reliable oxidative stress markers, were significantly increased in Long-MTX treated rats suggesting that MTX-induced kidney injury may be mediated through an increase in oxidative stress [168].
Dermatologic	Nodulosis (rare); Alopecia; Rash; Anaphylactic reactions	MTX-induced nodulosis may be mediated by adenosine through the adenosine A1 receptor [169]. MTX was shown to induce the generation of multinucleated giant cells, as does adenosine A1 receptor occupancy. This effect of MTX was reversed by a specific adenosine A1 receptor antagonist. Alopecia related to MTX treatment seems to be related to folate antagonism. In low dose MTX treatment, alopecia is rare and generally resolves several months after discontinuation [17,170].
Central nervous system (CNS)	Lethargy and fatigue; Headache, vertigo (less frequent)	Neurotoxicity of MTX may be related to MTX induced adenosine release and accumulation in the CNS. By acting at the A1 receptor on the perifornical lateral hypothalamus, adenosine may regulate wakefulness and somnolence and so potentially explaining asthenia and sleepiness experienced by some patients after MTX intake [171,172]. Other possible mechanisms of MTX-induced neurotoxicity are increased homocysteine levels and their excitatory amino acid neurotransmitter metabolites as homocysteic acid and cysteinesulfinic acid [173] and impairment of biopterin regerenating system in the brain, resulting in a reduced monoamine neurotransmitters availability [174].
Urogenital	Teratogenecity; oligospermia; gynecomastia (rare)	Use of MTX should be avoided before or during pregnancy because of its documented embryotoxicity and teratogenicity [175].
Musculoskeletal	Osteopathy; Osteoporosis	The effect of low dose MTX on bone was described in rats. Prolonged administration of low dose MTX in rats caused significant osteopenia with reduced osteoblast activity and increased osteoclast recruitment, which results in increased bone resorption [176]. However, no detrimental impact of MTX on the skeleton has been reported in patients treated with low dose MTX. MTX seems to have no clinically significant effect on bone mineral density (BMD) or on the osteoblast lineage [177,178].
Immunologic	Opportunistic infections	There is a belief amongst rheumatologists that MTX, as an immunosuppressant drug, is asssociated with the development of opportunistic infections. Weekly low-doses MTX can affect T cell activity [58], and cases of Pneumocystis pneumonia, nocardiosis, aspergillum, cryptococcosis, herpes zoster, herpes simplex and listeria-meningitis have been reported [170,179]. Despite some studies suggesting an increased risk of infection with MTX [180,181], several other studies have found that low-dose MTX does not appear to significantly increase the risk of infections in RA patients [182,183,184,185]. This risk appears to be associated with disease activity, comorbidities (diabetes, alcoholism) and the use of glucocorticoids, but not directly with MTX treatment [182]. It is well recognized that RA patients have significant increased risk of infection possibly due to chronic immune activation and inflammation which may impair immune function [185]. An increased risk of infection associated with MTX is possibly offset by the improvement of the immunological function secondary to the control of inflammation [185].
Others	Lymphoproliferative disorders	Lymphoproliferative disorders occur with increased frequency in RA patients compared to the general population, especially in the setting of high disease activity [170,184,186,187]. A relationship between MTX treatment and the occurrence of lymphoproliferative disorders in RA has been suggested. Long-term MTX therapy was associated with Epstein–Barr virus-related lymphoproliferative disorders with spontaneous regression after MTX withdrawal [188]. Despite its association with Epstein–Barr-associated lymphomas, there is currently no clear evidence that MTX provides additional risk of lymphoproliferative disorders to that of RA itself [184,189].
